# Supplementary material for: Similarities in Virulence and Extended Spectrum Beta-Lactamase Gene Profiles among Cefotaxime-Resistant Escherichia coli Wastewater and Clinical Isolates
Source: Antibiotics (Basel). 2022 Feb 17;11(2):260. doi: 10.3390/antibiotics11020260 (PMC8868091; doi:10.3390/antibiotics11020260)
Supplement: Supplementary file 1 [file antibiotics-11-00260-s001.zip › antibiotics-1593139-supplementary.pdf]

Supplementary Materials

Table S1. Primers used in this study.

Gene	Primer	Sequence (5′–3′)	Size (bps)	Tm (°C)	Reference
chuA	Forward	ATGGTACCGGACGAACCAAC	288	59	[25]
	Reverse	TGCCGCCAGTACCAAAGACA			
yjaA	Forward	CAAACGTGAAGTGTCAGGAG	211	59	[25]
	Reverse	AATGCGTTCCTCAACCTGTG			
TspE4.C2	Forward	CACTATTCGTAAGGTCATCC	152	59	[25]
	Reverse	AGTTTATCGCTGCGGGTCGC			
arpA	Forward	AACGCTATTCGCCAGCTTGC	400	59	[25]
	Reverse	TCTCCCCATACCGTACGCTA			
arpAgpE	Forward	GATTCCATCTTGTCAAAATATGCC	301	57	[25]
	Reverse	GAAAAGAAAAAGAATTCCCAAGAG			
trpA	Forward	AGTTTTATGCCCAGTGCGAG	219	59	[25]
	Reverse	TCTGCGCCGGTCACGCCC			
CTX-M-1 group	Forward	GGTTAAAAAATCACTGCGGC	863	56	[91]
	Reverse	TTGGTGACGATTTTAGCCGC			
CTX-M-2 group	Forward	ATGATGACTCAGAGCATTCG	865	56	[91]
	Reverse	TGGGTTACGATTTTCGCCGC			
CTX-M-9 group	Forward	ATGGTGACAAAGAGAGTGCA	863	56	[91]
	Reverse	CCCTTCGGCGATGATTCTC			
bla_{TEM}	Forward	CCGTGTCGCCCTTATTCC	786	60	[92]
	Reverse	AGGCACCTATCTCAGCGA			
bla_{SHV}	Forward	GGTTATGCGTTATATTCGCC	867	60	[93]
	Reverse	TTAGCGTTGCCAGTGCTC			
bla_{KPC1-5}	Forward	CATTCAAGGGCTTTCTTGCTGC	538	55	[94]
	Reverse	ACGACGGCATAGTCATTTGC			
bla_{OXA-1}	Forward	ATATCTCTACTGTTGCATCTCC	619	54	[95]
	Reverse	AAACCCTTCAAACCATCC			
Int1 (5′CS, 3′CS)	Forward	GGCATCCAAGCAGCAAG	variable	55	[96]
	Reverse	AAGCAGACTTGACCTGA			
fimH	Forward	AACAGCGATGATTTCCAGTTTGTGTG	465		[70]
	Reverse	ATTGCGTACCAGCATTAGCAATGTCC			
papC	Forward	GACGGCTGTACTGCAGGGTGTGGCG	328		[70]
	Reverse	ATATCCTTTCTGCAGGGATGCAATA			
sfa	Forward	CTCCGGAGAACTGGGTGCATCTTAC	410		[70]
	Reverse	CGGAGGAGTAATTACAAACCTGGCA			
afaC	Forward	CGGCTTTTCTGCTGAACTGGCAGGC	672		[70]
	Reverse	CCGTCAGCCCCCACGGCAGACC			
hlyca	Forward	AGATTCTTGGGCATGTATCCT	556		[70]
	Reverse	TTGCTTTGCAGACTGTAGTGT			
cnf	Forward	TTATATAGTCGTCAAGATGGA	693		[70]
	Reverse	CACTAAGCTTTACAATATTGA			
Aer (iucC)	Forward	AAACCTGGCTTACGCAACTGT	269		[70]
	Reverse	ACCCGTCTGCAAATCATGGAT			

Table S2. Antimicrobial susceptibility profiles present in *E. coli* isolates from different environments.

Resistance pattern	ESBL producer	Source			
		Clinic (n=11)	Hospital WW (n=22)	Urban Influent WW (n=19)	Urban Effluent WW (n=17)
No resistance cephalosporins, but ESBL producer		1			
ERT	N	1			
CAZ	N				1
CRO CTX	N				1
CRO CAZ CTX	N		2		2
CRO CAZ CTX ERT	N			2	
CRO CTX	Y			2	2
CRO CAZ CTX	Y				2
TET CRO CTX	N				1
TET CRO CAZ CTX	Y	2			
CIP CRO CTX	Y	1			
CIP CRO CAZ CTX	Y			1	
CIP TET CRO CTX	Y	1			
GEN TET CRO CAZ CTX	Y				1
GEN CIP TET CRO CAZ CTX ERT	N			1	
CHL SXT TET CRO CAZ CTX	Y				1
CHL SXT CIP TET CRO CTX	N	1			
CHL SXT GEN TET CRO CTX	Y			2	
CHL SXT GEN TET CRO CAZ CTX	Y				1
SXT TET CRO CAZ CTX	N			1	1
SXT TET CRO CTX	Y			2	
SXT TET CRO CAZ CTX	Y				1
SXT CIP CRO CTX	Y	2			
SXT CIP TET CRO CTX	N				1
SXT CIP TET CRO CTX	Y	1			1
SXT CIP TET CRO CAZ CTX	Y	1			1
SXT GEN TET CRO CTX	Y				2
SXT GEN TET CRO CAZ CTX	N			1	
SXT GEN TET CRO CAZ CTX	Y			2	
SXT GEN CIP CRO CAZ CTX	Y	1			
SXT GEN CIP TET CRO CAZ CTX	N		8	3	
SXT GEN CIP TET CRO CAZ CTX	Y		3	1	
SXT GEN CIP TET CRO CAZ CTX ERT	N			1	
SXT GEN CIP TET CRO CAZ CTX MEM	N		1		
SXT GEN CIP TET CRO CAZ CTX MEM ERT	N		3		
SXT GEN CIP TET CRO CAZ CTX MEM ERT	Y		1		
SXT GEN CIP TET CRO CAZ CTX MEM IMP ERT	N		2		
SXT GEN CIP TET CRO CAZ CTX MEM IMP ERT	Y		2		

Table S3. Phylogroups and sources of CTX-resistance.

Source	Prevalence (%)										
	N	A	B1	B2	C	D	E	Clade III, IV, or V	E or Clade 1	F	Unknown
Clinical	11	0	2 (18)	7 (64)	0	1(9)	0	0	0	1 (9)	0
Hospital WW	22	17 (77)	0	0	0	1(5)	1 (5)	3(14)	0	0	0
Urban Influ-ent	15	2 (13)	2 (13)	1 (7)	2 (13)	2 (13)	3 (20)	0	1 (7)	0	2 (13)
Treated ef-fluent	16	4 (27)	5 (33)	2 (13)	0	0	3 (20)	1 (7)	0	0	0
**Total**	63	23 (37)	9 (14)	10 (16)	2 (3)	4 (6)	7 (11)	4 (6)	1 (2)	1 (2)	2 (3)

Table S4. Plasmid-mediated antibiotic resistant phenotypes.

Strain	Antibiotic Susceptibility of Transformed Plasmids								
	CHL	SXT	GEN	CIP	TET	CRO	CTX	CAZ	MEM
Hospital WW									
1000C-C3	S	S	S	S	S	R	R	R	R
1000C-C4	S	S	S	S	S	R	R	R	R
1000D-C3	S	S	S	S	S	R	R	R	S
1000D-C4	S	S	S	S	S	R	R	R	R
WW influent									
I-C3	S	S	S	S	S	R	R	R	S
I-C10	S	S	S	S	S	R	R	R	S
WW effluent									
E-C3	S	S	S	S	S	R	R	R	S
E-C4	S	S	S	S	S	R	R	R	S
E-C5	S	S	S	S	S	R	R	R	S
E-C9	S	S	S	S	S	R	R	R	S

S: susceptible; R: resistant

Table S5. PlasmidFinder incompatibility groupings of *E. coli* 1000C-3.

**Organism and plasmid**	**Incompata-bility group**	**Identity (%)**	**Query / Tem-plate length**	**Position in con-tig**	**Accession number**
**pMHW-1**					
Serratia marcescens strain RIO-5 plasmid pRIO-5	IncP6	99.88	806 / 806	279..1084	JF785550
**pMHW-2**					
Escherichia coli K-12 plasmid F	IncFIA	99.48	388 / 388	120085..120471	AP001918
Escherichia coli K-12 plasmid F	IncFIB	98.51	603 / 682	1..603	AP001918
Escherichia coli plasmid pC15-1a	IncFII	95.42	262 / 261	58486..58747	AY458016

Table S6. Annotation of *E. coli* 1000C-3 plasmids.

Gene	Function	BLASTp Query Length	Query Coverage	% Identity	Accesssion	Location (nt)
**pMHW-1**						
**ARGs**						
merP	Mercury periplasmic binding protein	91	100%	100.00%	WP_003150552.1	6737..7012
merT	Mercury transport protein	116	100%	100.00%	WP_003089115.1	7025..7375
merR	Mercury resistance operon regulator	132	100%	100.00%	WP_003089120.1	9787..10185
KPC-2	Carbapenemase	293	100%	100.00%	WP_004199234.1	23393..24274
TEM-150	Extended-spectrum beta-lactamases	864	100%	99.54%	WP_045286946.1	24477..25363
**Plasmid Replication and Transfer**						
RIP	Replication Initiation Protein	458	100%	100.00%	WP_033996214.1	1..1377
KfrA	DNA-binding Protein	303	100%	100.00%	WP_033996289.1	10759..11670
DnaC	DNA helicase loader	251	100%	100.00%	WP_115455360.1	13662..14417
Relaxase	Mobilization Protein	890	100%	100.00%	CAE6190507.1	18532..21204
TnpR	resolvase	185	100%	100.00%	WP_001217881.1	26305..26862
TnpA	transposase	136	100%	100.00%	WP_025760448.1	27025..27435
Xho1	Restriction Enzyme	170	100%	99.41%	CAE6310107.1	37821..38333
ParA	plasmid partitioning	212	100%	100.00%	WP_033996216.1	38775..39413
**pMHW-2**						
**ARGs**						
dfr17	Trimethoprim resistance	210	100%	100.00%	AIG72712.1	14653..14681
aadA5	Streptomycin resistance	262	100%	100.00%	WP_000503573.1	15530..16318
qacE	Quaternary ammonium efflux transporter	115	100%	100.00%	WP_000679427.1	16524..16871
sul1	Sulfonamide resistance	279	100%	99.64%	WP_060614905.1	16865..17704
mphR	Macrolide resistance	194	100%	100.00%	AHX99591.1	21196..21780
mrxA	Macrolide resistance	412	100%	99.76%	WP_053919431.1	21780..23018
mph(A)	Macrolide resistance	301	100%	99.67%	EFY0167116.1	23015..23920
CTX-M-15	Extended-spectrum beta-lactamases	291	100%	100.00%	WP_000239590.1	27443..28318
catB3	Chloramphenicol resistance	182	100%	100.00%	WP_012783949.1	29577..30125
OXA-1	Extended-spectrum beta-lactamases	291	100%	100.00%	CAE6180605.1	30261..31091
aac(6′)-lb-cr	Aminoglycoside resistance	230	100%	99.57%	ACQ41894.1	31222..31821
tetR	Tetracycline repressor	82	100%	100.00%	EAV8592204.1	34517..34765
tetR	Tetracycline repressor	142	100%	100.00%	VCZ91928.1	34765..35193
tetA	Tetracycline resistance	246	100%	100.00%	WP_080393458.1	35730..36470
sitD	Hydrogen peroxide resistance; iron ABC transporter	285	100%	100.00%	WP_000968139.1	139015..139872
**Virulence gene**						
iutA	Receptor ferric arerobactin	733	100%	100.00%	WP_000973519.1	127712..129913
iucD	Biosynthesis of siderophore aerobactin	425	100%	100.00%	WP_000750130.1	129995..131272
iucC	Biosynthesis of siderophore aerobactin	580	100%	100.00%	WP_001015721.1	131269..133011
iucB	Biosynthesis of siderophore aerobactin	315	100%	100.00%	WP_000011908.1	133011..133958
iucA	Biosynthesis of siderophore aerobactin	574	100%	100.00%	WP_000602863.1	133959..135683
**Plasmid maintenance**						
pemK	Endoribonuclease (toxin)	110	100%	100.00%	WP_000439434.1	42421..42822
pemI	Anti-toxin	85	100%	100.00%	WP_000557619.1	42755..43012
ltrA	Group II intron reverse transcriptase	633	100%	100.00%	WP_000936285.1	104447..106348
sopB	Control of plasmid partitioning	323	100%	100.00%	WP_000817031.1	116851..117822

sopA	Control of plasmid partitioning	388	100%	100.00%	WP_000772446.1	117822..118997
repE	Replication initiator	251	100%	100.00%	WP_000852146.1	119576..120331
ccdB	Toxin: inhibitor of DNA topoisomerase type II	101	100%	100.00%	WP_001159871.1	121860..12265
ccdA	Anti-toxin: inhibits post-segregational killing by ccdB	72	100%	100.00%	WP_000813630.1	122167..122385
**Tra operon**						
TraX	Pilus Acetylase	248	100%	100.00%	WP_023149624.1	64678..65424
TraI	Helicase	1706	100%	100.00%	WP_013362805.1	65444..70564
TraD	Transfer Coupling Protein	729	100%	100.00%	WP_040110343.1	70564..72753
TraT	Complement Resistance Protein	243	100%	100.00%	WP_000850424.1	73005..73736
TraS	Transfer exclusion protein	169	100%	100.00%	WP_000628100.1	73750..74259
TraG	Transfer Protein	654	98%	99.07%	EEY5833906.1	75941…77905
TraH	Transfer Protein	458	100%	100.00%	WP_013362802.1	77902…79278
TrbF	Transfer protein	130	100%	100.00%	WP_000164675.1	79262…79654
TrbJ	Transfer protein	104	100%	100.00%	WP_152932543.1	79608..79992
TrbB	Pilin Assembly Protein	184	100%	100.00%	WP_001553830.1	79912..80466
TraQ	Pilin Chaperone	89	100%	94.68%	WP_000624105.1	80453..80722
TrbA	Transfer protein	113	100%	100.00%	WP_000556794.1	81119..81460
TraF	Pilin Assembly Protein	247	100%	99.60%	WP_062894221.1	81474..82217
TrbE	Transfer protein	86	100%	100.00%	ARZ86200.1	82210..82470
Trbu	Transfer protein	86	100%	100.00%	ARZ86200.1	82210..82470
TraN	Stabilization Protein	602	100%	100.00%	WP_000821840.1	82494..84302
TrbC	Pilin Assembly Protein	208	100%	100.00%	PVF70563.1	84299..84925
TraU	Pilus Assembly Protein	330	100%	100.00%	WP_000830180.1	84946..85938
TraW	Transfer Protein	210	100%	100.00%	WP_001203720.1	85935..86567
TrbI	Transfer protein	128	100%	100.00%	WP_000099686.1	86564..86950
TraC	Type IV secretion	875	100%	99.89%	WP_001064245.1	86947..89574
TraR	Dksa	73	100%	100.00%	CAF2485283.1	89734..89955
TraV	Lipoprotein	171	100%	100.00%	WP_000809838.1	90090..90605
TrbG	Transfer protein	83	100%	100.00%	WP_001038342.1	90602..90853
TraB	Transfer Protein	475	100%	100.00%	WP_001825178.1	91629..93056
TraK	Secretin	242	100%	100.00%	WP_001230787.1	93056..93784
TraE	Transfer Protein	188	100%	100.00%	WP_000399792.1	93771..94337
TraL	Transfer Protein	103	100%	100.00%	WP_000012106.1	94359..94670
TraA	Pilin	121	100%	100.00%	WP_000994779.1	94685..95050
TraY	Relaxosome	131	100%	100.00%	CAE6395130.1	95083-95478
TraJ	conjugal transfer transcriptional activator	229	100%	100.00%	WP_000283385.1	95577..96266
TraM	Relaxosome	127	100%	100.00%	WP_001151524.1	96453..96836

Table S7. BLASTN results of nucleotide homology to *E. coli* 1000C-3 plasmids.

Organism	Query cover (%)	Identity (%)	E-value
**pMHW-1**			
Pseudomonas aeruginosa strain 10265 plasmid p10265-KPC, complete sequence	88%	99.95%	0
Citrobacter braakii strain CRE3 plasmid pCRE3-KPC, complete sequence	62%	99.95%	0
Klebsiella pneumoniae strain A1705 plasmid pA1705-KPC, complete sequence	89%	99.89%	0
Citrobacter freundii isolate CF121SC21 genome assembly, plasmid: 1	89%	99.89%	0
Enterobacter hormaechei strain 189 plasmid pECL189-1, complete sequence	62%	99.89%	0
Aeromonas veronii plasmid pWP8-W19-CRE-03_2 DNA, complete genome, strain: WP8-W19-CRE-03	89%	99.89%	0
Aeromonas hydrophila plasmid pWP8-S18-ESBL-02_1 DNA, complete genome, strain: WP8-S18-ESBL-02	89%	99.89%	0
Aeromonas hydrophila subsp. hydrophila strain WCHAH045096 plasmid pKPC2_045096, complete sequence	89%	99.88%	0
Enterobacter cloacae strain 30860 plasmid p30860-KPC, complete sequence	89%	99.87%	0
Enterobacter cloacae plasmid pWP8-W19-CRE-02_3 DNA, complete genome, strain: WP8-W19-CRE-02	88%	99.88%	0
Klebsiella pneumoniae strain A1706 plasmid pA1706-KPC, complete sequence	89%	99.88%	0
Klebsiella oxytoca strain pKOX3 plasmid p5-KPC	89%	99.86%	0
Enterobacter kobei strain WCHEK045523 plasmid pKPC2_045523, complete sequence	89%	99.85%	0
Aeromonas caviae plasmid pWP8-S18-CRE-01_2 DNA, complete genome, strain: WP8-S18-CRE-01	86%	99.85%	0
Klebsiella quasipneumoniae strain WW-14A plasmid pWW14A-KPC2, complete sequence	89%	99.75%	0
Aeromonas hydrophila GSH8-2 plasmid pGSH8-2 DNA, complete genome	89%	99.88%	0
Aeromonas caviae GSH8M-1 plasmid pGSH8M-1-2 DNA, complete genome	86%	99.84%	0
Aeromonas taiwanensis strain L186 plasmid p186-KPC, complete sequence	89%	99.66%	0
Citrobacter freundii plasmid pCRE12-KPC, complete sequence	70%	100.00%	0
Citrobacter sp. strain 172116965 plasmid p116965-KPC, complete sequence	87%	100.00%	0
Aeromonas sp. ASNIH3 plasmid pKPC-cd17, complete sequence	89%	99.84%	0
Escherichia coli strain Ecol_542 plasmid pEC542_KPC, complete sequence	89%	100.00%	0
**pMHW-2**			
Escherichia coli strain EC45 plasmid pEc45_1, complete sequence	89%	99.86%	0
Escherichia coli strain SCU-120 plasmid pSCU-120-1, complete sequence	95%	99.77%	0
Escherichia coli strain E-T67-1 plasmid punnamed1, complete sequence	87%	99.83%	0
Escherichia coli str. 3249 plasmid RCS105_pI, complete genome	100%	99.74%	0
Escherichia coli YJ3 plasmid pYJ3-a DNA, complete genome	94%	99.85%	0
Escherichia coli E308 plasmid pE308_IMP6 DNA, complete sequence	83%	99.84%	0
Escherichia coli isolate EC-TO75 genome assembly, plasmid: 2	67%	99.81%	0
Escherichia coli strain M63c plasmid pMB2, complete sequence	82%	99.73%	0
Escherichia coli strain CA14 plasmid pCA14, complete sequence	90%	99.83%	0
Escherichia coli strain p11A plasmid p11A_p2, complete sequence	87%	99.81%	0
Escherichia coli strain F16EC0507 plasmid pF16EC0507-1, complete sequence	72%	99.81%	0
Escherichia coli strain F17EC0098 plasmid pF17EC0098-1, complete sequence	79%	99.81%	0
Escherichia coli strain C17EC0083 plasmid pC17EC0083-1, complete sequence	86%	99.81%	0
Escherichia coli strain F16EC0121 plasmid pF16EC0121-2, complete sequence	75%	99.81%	0
Escherichia coli strain D16EC0206 plasmid pD16EC0206-2, complete sequence	87%	99.81%	0
